# Tracking progress to 2030

**DOI:** 10.2471/BLT.15.030715

**Published:** 2015-07-01

## Abstract

Pali Lehohla tells Fiona Fleck why more tools are in place to measure progress towards the next set of health and development goals.

Q: What have been the challenges for tracking health and development progress towards the Millennium Development Goals (MDGs) that end this year?

A: The MDGs were very top down. We statisticians were confronted with decisions made by heads of state at the Millennium Conference in 2000 and asked to measure progress towards those goals. We were not consulted beforehand on what should be measured and had to develop measurement tools with little or no additional resources. As a result of the MDG project many countries have enacted statistical reforms. My country did so in 1999, as part of the transformation after the end of apartheid in 1994. Measurement – the work of statisticians – often gets forgotten in processes of change and once all the dutiful work is done, the politicians ask us: “How well have we done?” and we say: “But you didn’t share with us upfront what you wanted us to measure. Statistical processes take time.”

“Measurement – the work of statisticians – often gets forgotten.”

Q: Countries are set to gather at the United Nations in New York in September to agree on 17 new development goals between 2015 and 2030, the Sustainable Development Goals (SDGs). Of 169 targets proposed, 13 are related to health. Were you consulted this time?

A: Yes. I am one of a group of statisticians from many countries asked by the United Nations to come up with indicators for the new goals and targets. We aim to provide some tentative indicators in September, when the goals are to be adopted by countries, and deliver our final proposals in March 2016. It’s an incredibly complex and difficult task, as I told the United Nations Open Working Group on the SDGs, they should not thrust greatness upon us – we need more gentle courting.

Q: Why so complex and difficult?

A: We need to gain much more “granularity” (detail) and to do so takes time. We can achieve this partly with population censuses, but many low- and middle-income countries do not hold these regularly. Without benchmarks and data points updated regularly over time, measurement is virtually impossible. We need good quality, reliable and detailed data aggregated by age and sex to measure progress toward the health goals. The best way to obtain these data is for countries to establish sound civil registration and vital statistics systems that record and track their populations throughout their lives.

Q: Is that already happening?

A: In the African Symposium on Statistical Development we have ignited a civil registration and vital statistics movement, and we are currently doing assessments to see what is needed to establish such systems in African countries. In South Africa, the push for birth and death certification for the whole population was triggered by a change in the law in 1998 and resulted in our civil registration and vital statistics system involving the ministries of home affairs and health and Statistics South Africa. The problem is compliance.

Q: Why?

A: Collation of information requires an understanding of why this needs to be done and how data are used. When my cousin passed away in 2002, the doctor who certified his death omitted some details from the form and the cause of death was incorrect. I asked him why, and he said: “That’s not important, it’s just for statistics”. I was already the statistician-general of South Africa then and, despite my high office, had to choose between mourning two deaths or one: I chose my cousin’s death – not the death of statistics. Doctors need to know that we need these data to count, for example, the deaths of women due to the complications of pregnancy and childbirth or the deaths of infants in their first year of life, and that this information is needed to make health plans and manage health programmes.

Q: It is estimated that only one in three deaths are recorded globally. How are other countries in Africa doing in terms of recording causes of death?

A: Apart from South Africa, Algeria, Botswana, Mauritius, Morocco, Seychelles and Tunisia are among a few African countries that record causes of death in ways that are classified as comprehensive.

Q: You managed censuses for Bophuthatswana, in South Africa in the 1980s, and led work on the South African Census in 1996. How useful are data from censuses for measuring progress towards the post-2015 goals?

A: Yes, I cut my teeth in a homeland census. My only regret is that the Bophuthatswana politicians didn’t think the data were important and hardly used them. It was an important experience. Bophuthatswana was a creation of the apartheid regime and our results differed dramatically from previous regime-led censuses which underlined the deep disregard for the black population. In 1996, we used the same techniques in the first comprehensive census of post-apartheid South Africa as we used in Bophuthatswana. Before that, censuses only adequately covered 10–12% of the population, the white population, leaving out the majority black population. The census of 1996 was a turning point, as President Nelson Mandela put it, the results revealed “a society which had enormous basic needs to be met, whether it be in terms of access to clean water; electricity, telephones or schooling. By measuring the extent of deprivation in October 1996, the results provide us with benchmarks against which our performance, as government and nation, should be measured year by year.”

Q: How many countries in Africa have such data?

A: As a result of aggressive advocacy by the African Symposium on Statistical Development, 49 of the 54 countries in Africa participated in the current 2010 round of Population and Housing Censuses. In general, 15 of the 54 countries have not held censuses regularly, often due to current or past conflicts and, by current standards, census data obtained every 10 years is not frequent enough for measuring progress towards development goals because you need interim data. Household surveys can provide such data, but are not conducted regularly with the required level of granularity. In South Africa, we measure mortality and the causes of death quite well, but at a granular level of maternal mortality, we still have difficulty understanding what exactly is killing women who are pregnant or have just given birth. That is why the most important thing for measuring health and development progress is to establish a civil registration and vital statistics system, because it gives you your denominators when births are registered and your numerators when deaths are certified.

Q: Will countries manage to build such systems in time to track progress from now until the year 2030?

A: Scandinavian countries took centuries to perfect their civil registration systems and many countries in Africa have not moved an inch towards creating useable civil registration and administrative records systems. We should not be intimidated. Programmes and actions are now under way to implement this, green shoots are now visible in Africa and Asia, where countries made a commitment to civil registration in 2014, and we are beginning to see dividends. Other data sources will be used to complement civil registration and vital statistics systems to measure progress towards the new development goals.

Q: What tools will help?

A: Portable devices and smartphone technologies are promising. In African countries, many people – even from low socioeconomic groups – own mobile phones, while the Internet is still mainly accessed by higher socioeconomic groups, so this is a route to reaching most of the population in low- and middle-income countries. Another major problem is the lack of addresses.

Q: Why?

A: We need to have street addresses to link every civil registration record with a place of abode. We need physical addresses to deliver services, such as water and sanitation, deal with emergencies better and gather information about the population for use in evidence-based policy-making. The challenge is in defining addresses in villages without streets or house numbers. In African countries, people say “the mud house next to the large tree”, but such descriptions are not fit for modern society. Africa must have addresses and we need to do this scientifically using latitude, longitude and altitude. In South Africa, about 30% of people don’t have proper addresses and it’s worse in many other African countries. In South Africa, we have designed a method to assign addresses to households without conventional addresses. It is a community-driven approach with methodological support from Statistics South Africa and this could be adopted in other countries.

“Without data and statistics we cannot be transparent and accountable, manage for results or achieve transformative change.”

Q: Who will measure progress towards the goals?

A: Measurement is everybody’s business and – paradoxically – no one’s business. Every individual or organization involved in measurement – whether local government officials or international experts – needs to comply with the scientific standards to determine whether progress is being achieved.

Q: The proposed 13 health targets are said to have some of the best measuring instruments available among the proposed post-2015 goals. How can this be?

A: At face value the health targets seem easy to measure. The doctor records the age, sex and other attributes of a patient, such as weight, temperature and blood pressure. So health records are among the most elaborate and precise. The problem is that health information systems are notoriously disjointed. Given the precision and detail of health records compiled in hospitals or clinics, deaths of pregnant women, for example, can be measured, children can be counted when they are born and their birth weight registered. Thus we know if a child is malnourished and we know whether someone has been diagnosed with tuberculosis or tested positive for HIV. The problem is that when the size of the catchment populations that serve as denominators is not known, the numbers have no context. When health records remain at the hospital and are not shared, they are of no use to the national health information system. Without data and statistics we cannot be transparent and accountable, manage for results or achieve transformative change.

